# 24-hr observation unit is safe location for rapid glucose control in uncomplicated severe hyperglycaemia

**DOI:** 10.1186/s12873-021-00460-0

**Published:** 2021-05-30

**Authors:** I. Ibrahim, R. Macatangay, C. Y. Chai, C. M. Khoo, M. Mahadevan

**Affiliations:** 1 Emergency Medicine Department, National University Hospital, Level 4 National University Centre of Oral Health, 9 Lower Kent Ridge Road, Singapore, 119085 Singapore; 2 Department of Surgery, Yong Loo Lin School of Medicine, Singapore, Singapore; 3 Department of Medicine, National University Hospital, Singapore, Singapore; 4 Yong Loo Lin School of Medicine, Singapore, Singapore

**Keywords:** Observation unit, Diabetes mellitus, Hyperglycaemia, Hypoglycaemia

## Abstract

**Background:**

Uncomplicated hyperglycaemia is a common presentation in the emergency department (ED). Rapid glucose control is associated with the risk of iatrogenic hypoglycaemia. We sought to determine the safety of a rapid glucose control protocol delivered in a 24-h emergency department observation unit (OU).

**Methods:**

This is a retrospective chart review of patients admitted to the OU for hyperglycaemia where the assessing clinician deemed there was no other reason for medical admission apart from hyperglycaemia; and that the patient could be safely discharged provided their hyperglycaemia was adequately treated. The rapid glucose control protocol consists of 4–6 hourly glucose monitoring and insulin injections according to a sliding scale. We report the demographics, reduction in glucose values and the incidence of hypoglycaemia in the OU. We also determine the rate of discharge from OU and the rate of hospital admission at 30 days.

**Results:**

We included 101 patients. The mean age was 53.5 years (95% CI 50.4–56.6) and 64% of patients were male. The mean HbA1c value was 12.8% (95% CI 12.3–13.3). The mean admission and discharge glucose values were 27.2 (95% CI 26.3–28.1) and 13.9 (95% CI 13.2–14.6) mmols/l respectively. There was no incidence of hypoglycaemia in the OU. We successfully discharged 90.1% of the patients from the OU, of which 3 (3.3%) patients were admitted to the hospital within 30 days of discharge.

**Conclusion:**

ED OU is a safe location to deliver effective management for patients presented with uncomplicated severe hyperglycaemia.

## Background

Hyperglycaemia contributes as high as 20% of patients presenting to the Emergency Department (ED) [1]. Most of the patients do not have hyperglycaemia emergencies such as diabetic ketoacidosis or hyperosmolar hyperglycaemia state [2]. Left untreated, chronic severe hyperglycaemia can accelerate microvascular and macrovascular complications leading to disabling retinopathies, nephropathies, neuropathies and cardiovascular disease [3, 4]. Patients presenting to the ED with acute hyperglycaemia but who are otherwise well may not need admission to the hospital, but require adequate treatment of their hyperglycaemia before they can be safely discharged.

Currently, there is no consensus in regard to the location in the ED where glucose control could be achieved safely, i.e. minimizing the risk of hypoglycaemia [5]. Typically, patients with hyperglycaemia would be admitted to the ward, resulting in an average ward length stay of 2–5 days [6]. They would have received glucose control and diabetes education during the stay. Ward admissions incur high healthcare costs and also disrupt patients’ daily self-management routines, but would typically provide an opportunity for multidisciplinary team education during the stay [7]. A recent study showed that patients with hyperglycaemia presented at the ED could be discharged directly from the ED with a modest glucose control (blood glucose at discharge of 17.6–18.6 mmol/L) [5]. About 2% of these patients developed iatrogenic hypoglycaemia during the ED stay of about 5 h. Another study showed < 1% incidence of iatrogenic hypoglycaemia with less stringent glucose control (blood glucose at discharge of 19.4–33.3 mmol/L) [8].

Weighing the need for optimal blood glucose control and minimizing the risk of iatrogenic hypoglycaemia, an observation unit (OU) would appear to be a reasonable location to deliver rapid care and ensure patient safety. Previous studies had either described the care of diabetic patients with hyperglycaemia in an OU for more than 2 days; or had not reported the incidence of iatrogenic hypoglycaemia [9–12]. The advantages of using OU as a location include a greater time margin to discharge the patient and having a dedicated team to care for the patients; as opposed to the ED, which frequently attends to concurrent emergencies. Furthermore, the OU is also a conducive place to introduce diabetes education through a multidisciplinary team. Here, we aimed to determine the efficacy and safety of a rapid glucose control protocol delivered in a 24-h OU.

## Methods

This is a retrospective cohort study conducted at the ED of a tertiary hospital from January 2014 to July 2016**.** The ED received more than 120,000 attendances per year. This study was approved by the National Healthcare Group Domain Specific Review Board (2016/01337). We focused on patients for whom the assessing clinician deemed that there was no other reason for medical admission apart from hyperglycaemia; and who it was felt could be safely discharged provided their hyperglycaemia was adequately treated. The inclusion criteria were: age 21 years and above, initial ED laboratory venous blood glucose level > 20 mmol/L but < 35 mmol and not in hyperglycemic crisis i.e. diabetic ketoacidosis or hyperosmolar hyperglycemic state. We excluded patients who were pregnant or had concurrent triggering factors such as acute myocardial infarction, cerebrovascular accident and sepsis.

### Glucose-lowering protocol in the OU

The OU is a 16-bed unit managed by emergency physicians 24 h a day. Upon entering the unit, the patients were put on the hyperglycaemia protocol. The protocols and choice of insulin were introduced following consultation with the diabetes team. All treatment with insulin or oral hypoglycaemic agents (OHA) were ceased except metformin. The patients were only allowed clear fluids and intravenous hydration. Capillary blood glucose levels were monitored 4 to 6 hourly. Subcutaneous soluble insulin administration was given according to an insulin sliding scale (Table 1). On the following day at breakfast, patients who are newly diagnosed with diabetes mellitus (DM) or patients who were insulin-naïve were started on premixed insulin. For patients who are already on insulin therapy before, the existing insulin dose was resumed and the dose was adjusted accordingly. Upon the discretion of the ED consultant, the multidisciplinary diabetes team review might be requested to see the patient before discharge. Patients were discharged from the OU when the glucose control was less than 20 mmol/L within 24 h of observation. They were given referral to either the primary health or specialist clinics upon discharge.

**Table 1:** Insulin sliding scale

Capillary blood glucose (mmol/L)	Subcutaneous soluble insulin (units)
10.1–14.0	4
14.1–18.0	6
18.1–24.0	8
> 24.1	10 (maximum)

### Outcomes

We recorded the incidence of hypoglycaemia during the OU stay [defined as blood glucose of ≤ 3.9mmols/L in our institution] and the disposition from the ED. [13] At 30 days, we determined if the patient was readmitted via chart reviews.

### Statistical analysis

Data were analyzed using the PASW Version 24.0 statistical software. Continuous variables were summarized as means and 95% CI while dichotomous and categorical data were summarized as frequencies and percentages.

## Results

During the 19-month period, 101 patients were admitted to the OU for hyperglycaemia. The mean age was 53.5 years (95% CI 50.4–56.6) and 64% were male (Table 2). All patients but one had Type 2 diabetes. In 26.7% of the patients, the ED attendance and subsequent admission to the OU were the first presentation of hyperglycaemia. For those already on diabetes treatment, 50.0% (37/74) and 82.4% (61/74) were on insulin and OHA respectively. Of those on OHA (*n* = 61), 49.2% (30/61) were on metformin, 41.0% (25/61) were on sulphonylureas, 4.9% (3/61) were on alpha-glucosidase inhibitors and 3% (2/61) were on dipeptidyl peptidase inhibitors. The mean HbA1C for 85 patients was 12.8% (95% CI 12.3–13.3).

**Table 2:** Description of overall cohort (*n* = 101)

Variables	Frequency n (%)
**Mean age** (years)	53.5 (95% CI 50.4–56.6)
**Male**	64 (63.4)
**Race**	
Chinese	43 (42.6)
Malay	39 (38.6)
Indian	15 (14.9)
Others	4 (4.0)
**Past history**	
Hyperlipidemia	48 (47.5)
Hypertension	53 (52.9)
Ischemic Heart Disease	8 (7.9)
Stroke	4 (4.0)
Acute Myocardial Infarction	3 (3.0)
**Duration of DM**	
New presentation	27 (26.7)
Less than 6 months	9 (8.9)
6 months to 1 year	7 (6.9)
1 to 5 years	17 (16.8)
5 to 10 years	21 (20.8)
10 years and above	20 (19.8)
**Current medications for known diabetics** (*n* = 74)	
Diet control only	1 (1.4)
OHA only	36 (48.6)
Insulin only	12 (16.2)
OHA + insulin	25 (33.7)
**Mean HbA1C** (%) (*n* = 85)	12.8 (95% CI 12.3–13.3)
**Mean glucose (mmols/L)**	
Admission	27.2 (95% CI 26.3–28.1)
Discharge	13.9 (95% CI 13.2–14.6)
**OU outcomes**	
Discharged	91 (90.1)
Admitted to the ward	10 (9.9)

*OU* observation unit, *OHA* oral hypoglycaemic agent

The mean admission and discharge blood glucose levels were 27.2 (95% CI 26.3–28.1) and 13.9 (95% CI 13.2–14.6) mmol/l respectively. The mean difference was 13.3 mmols/l (95%CI 12.3–14.3) (*p* < 0.001). There was no incidence of hypoglycaemia. The multidisciplinary diabetes team reviewed 86.1% (87/101) of the patients. In regard to disposition, 10 patients (9.9%) were admitted to the ward for various reasons such as skin infection that evolved to need surgical drainage while in the OU (*n* = 1), persistent hyperglycaemia with glucose level above 20 mmol/L (*n* = 7), hypotension during hemodialysis (*n* = 1) and social reasons (*n* = 1). For those who were discharged from the ED, the mean length of stay in OU was 16.9 h (95% CI 15.8–17.9). Among these patients, 14 patients had an increase in the diabetes medication doses while 54.9% (51/92) were prescribed with new medications. Within 30 days of OU admission, there were three patients (3.3%) readmitted to the ED. Two patients were readmitted for recurrence of severe hyperglycaemia and one patient for hypoglycaemia (Table 3).

**Table 3:** Description of patients readmitted to hospital at 30 days post-OU

Patient	Reason for admission	Description
1	Hyperglycaemia	Male/50 years
		• Multiple history of non-compliance
		• DM duration was 1–1.5 years. No HbA1c done
		• On OHA and insulin
		• Admission and discharge glucose was 33.3 and 10.6 mmols respectively
2	Hyperglycaemia	Male/52 years
		• DM duration less than 6 months. HbA1c 14%.
		• Admission and discharge glucose was 33.3 and 15.6 mmols respectively.
		• On OHA only and started on insulin in OU.
3	Hypoglycaemia	Male/66 years
		• Hypoglycaemia on the 26th day post-OU discharge
		• DM duration was 1 year. HbA1c 13.9%.
		• Admission and discharge glucose was 35.9 and 13.3 mmols respectively.
		• Previously on OHA only and was started on insulin in the OU.

*DM* diabetes mellitus, *OHA* oral hypoglycaemic agent, *OU* observation unit

## Discussion

Our study showed that patients who presented with severe uncomplicated hyperglycaemia but are otherwise well, the ED OU was a safe location to improve the blood glucose rapidly within 24 h. There was no incidence of hypoglycaemia in the OU. We achieved good blood glucose control as reflected by the mean discharge blood glucose of 13.9 mmol/L. In comparison, Driver’s et al. study showed that despite a higher mean discharge blood glucose of 18 mmol/l, 1.6% (9/566) of patients developed hypoglycaemia during ED stay of about 5 h [5]. In another study, 0.9% (1/110) of patients developed hypoglycaemia during ED stay of about 4 h but with a less stringent blood glucose control (discharge glucose of 19.4 to 33.3 mmol/L) [8]. These studies showed that the balance between the rapidity of glucose control and risk of hypoglycaemia was possibly involved in the outcomes. If the management aim was for both safety and adequate blood glucose control, a less aggressive approach might be needed to manage these patients but this translates to a longer length of stay.

A study by Crilly et al. described a similar 24-h OU experience in managing patients with hyperglycaemia with regards to average age, chronicity of poorly controlled diabetes as reflected by the admission HbA1c and blood glucose achieved at discharge [12]. However, our study had a higher proportion of patients with initial blood glucose value above the 22.2 mmol/L (400 mg/dl). This observation may suggest that our study instituted a more rapid glucose lowering protocol to achieve the same discharge glucose. The study also reported no incidence of hypoglycaemia in the OU.

Proponents for ED as a location to manage well hyperglycaemic patients may argue that less stringent control can be a strategy to reduce the use of hospital beds in the ward or OU [5]. However, these studies had used a short period of 7 days to detect adverse outcomes which may indicate that patients will need early follow-up to manage their blood glucose. Such a strategy is suitable to healthcare systems with efficient follow-up infrastructure. In a recent systematic review of the factors and reasons associated with non-attendance in follow up patients with diabetes mellitus, healthcare provider factors such as scheduling, duration between appointments were associated with non-attendance [14].

We have shown that the observation unit is a safe location to control blood glucose in patients with hyperglycaemia. However, safe practices in the managing such patients should include multidisplinary diabetic team assessment and proper follow-up to maintain glycaemic control and hence obviates morbidity. The ED attendance in itself provides a golden opportunity to encourage a patient to re-engage with his/her own diabetes care. Hence, future observation unit protocols for patients with hyperglycaemia should incorporate obligatory multidisciplinary team review and robust follow-up strategies.

### Limitations

Firstly, this is a single-center study and hence the results may not be replicable to all OUs. However, we believe that there are no special resources needed to run this protocol, and hence it can be adapted to other settings. Secondly, the ED reattendances and readmission rates might be underestimated as patients might attend other hospitals.

## Conclusions

Patients with uncomplicated hyperglycemia can be effectively and safely managed in the ED OU with a clear hyperglycemia management protocol. This strategy reduces hospital admissions and potentially reduces the health care cost without impacting on patient’s outcomes.

## Data Availability

The datasets used and/or analysed during the current study are available without personifying information from the corresponding author on reasonable request.
